# Efficient strategies for finger movement classification using surface electromyogram signals

**DOI:** 10.3389/fnins.2023.1168112

**Published:** 2023-06-22

**Authors:** Sunil Kumar Prabhakar, Dong-Ok Won

**Affiliations:** Department of Artificial Intelligence Convergence, Hallym University, Chuncheon, Republic of Korea

**Keywords:** EA, BBN, ELM, FCM, EWT, LMD, LS-SVM

## Abstract

One of the famous research areas in biomedical engineering and pattern recognition is finger movement classification. For hand and finger gesture recognition, the most widely used signals are the surface electromyogram (sEMG) signals. With the help of sEMG signals, four proposed techniques of finger movement classification are presented in this work. The first technique proposed is a dynamic graph construction and graph entropy-based classification of sEMG signals. The second technique proposed encompasses the ideas of dimensionality reduction utilizing local tangent space alignment (LTSA) and local linear co-ordination (LLC) with evolutionary algorithms (EA), Bayesian belief networks (BBN), extreme learning machines (ELM), and a hybrid model called EA-BBN-ELM was developed for the classification of sEMG signals. The third technique proposed utilizes the ideas of differential entropy (DE), higher-order fuzzy cognitive maps (HFCM), empirical wavelet transformation (EWT), and another hybrid model with DE-FCM-EWT and machine learning classifiers was developed for the classification of sEMG signals. The fourth technique proposed uses the ideas of local mean decomposition (LMD) and fuzzy C-means clustering along with a combined kernel least squares support vector machine (LS-SVM) classifier. The best classification accuracy results (of 98.5%) were obtained using the LMD-fuzzy C-means clustering technique classified with a combined kernel LS-SVM model. The second-best classification accuracy (of 98.21%) was obtained using the DE-FCM-EWT hybrid model with SVM classifier. The third best classification accuracy (of 97.57%) was obtained using the LTSA-based EA-BBN-ELM model.

## 1. Introduction

Performing daily activities without a forearm is quite difficult for people who have suffered the loss of it. Individuals whose upper and lower limbs have been amputated experience a lot of physical and mental trauma as they cannot perform their daily activities (Ouyang et al., 2014). Some kind of a prosthetic device is required by these individuals so that their daily activities can be adequately completed. When the muscle movement is experiencing some kind of disruption, these prosthetic devices are highly useful to tackle it (Su et al., 2020). In order to control prosthetic limbs such as wrists and hands, one widely used signal is EMG (Gokgoz and Subasi, 2015). The recordings of the EEG signals originating from the specific muscles related with hand and finger gestures are utilized to control and administer many types of movements. The classification of individual finger gestures are more difficult to perform than the classification of the whole hand as the usage of muscles for individual finger movement is quite complex in nature. In order to assess the presence of nerve dysfunction, disruption in the neuromuscular signal transmission, and muscle dysfunction, EMG is utilized (Sezgin, 2015). Furthermore, it is also widely used for diagnosing various kinds of chronic pains in the lower back and head. A set of multiple motor unit action potentials are superimposed upon each other to form an EMG. The resulting signal is explained in the terms of frequency, amplitude, and phase as a function of time and it is usually stochastic in nature. The categorization of the EMG signals is done depending on how they are acquired from the body, whether in an invasive manner or non-invasive manner (Jali et al., 2014). With the advent of machine learning techniques, EMG signals are used to detect human activities and movements. Once this knowledge is acquired, it is implemented fully inside robots so that those activities can be replicated. Some of the famous works undertaken on EMG signal classification with machine learning techniques are reported as follows.

A novel finger movement classification technique dependent on multi-centered binary pattern (MCBP) using EMG signals was reported in Tuncer et al. (2022), where high classification accuracies of 99.17, 99.7, and 99.62% were obtained for three different cases. The EMG finger movement classification was implemented using an adaptive neuro fuzzy inference system (ANFIS) which proved that the classification of finger gestures is less than the classification of the hand gestures (Caesarendra et al., 2018). The high-density EMGs of intrinsic and extrinsic hand muscles for exploring finger movement decoding was repeated in Hu et al. (2022). Artificial Neural Networks (ANNs) were used for the EMG-based classifications of hand and finger gestures, reporting a mean accuracy of 0.940 (Lee et al., 2021). A high-precision wireless surface EMG sensor was developed for finger gesture recognition using the sensing and classification of EMG signals (Jianting et al., 2021). An interesting method of utilizing cross recurrence plots was implemented in EMG hand movement recognition (Aceves-Fernandez et al., 2019). A multichannel Convolutional Neural Networks (CNN) was also used for the heterogenous hand guise classification depending on the Surface Electromyogram (sEMG) signals (Sikder et al., 2019). Two-channel surface Electromyogram (EMG) signals were utilized to classify the hand and finger movements with ELM classifiers reporting an accuracy of 98.95% (Sezgin, 2019). A fractal-based classification of EMG signals was performed where the results analysis showed that EMG signals have the greatest fractal dimension in the case of thumb extension and the lowest fractal dimension in the case of little finger extension (Namazi, 2019). The classification of five-finger movement depending on a low-cost, real-time EMG system was developed in Seguna et al. (2020). Sparse filtering of wavelet packet coefficients was used for EMG-based finger movement recognition, reporting an accuracy of 99.52% (Bhagwat and Mukherji, 2020). A deep learning model that combines a convolutional auto-encoder and convolutional neural network (CAE + CNN) for classifying an EMG data set comprising 10 classes of hand gestures was reported with an accuracy of 99.38% (Jia et al., 2020). A particle swarm optimization-based support vector machine (PSO-SVM) algorithm was used for the classification of finger movements using EMG signals with a success of pattern recognition between 68 and 86% (Pamungkas et al., 2022). The proportional estimation of finger movements from high-density sEMG was conducted and the common spatial patterns proportional estimation (CSP-PE) outperformed the linear discriminant analysis (LDA; Celadon et al., 2016). Different limb positions were used for the evaluation of feature projection techniques in object grasp classification via EMG signals using a spectral regression ELM technique (Thiamchoo and Phukpattaranont, 2022). Hybrid CNN-SVM architecture was used for the classification of EMG signals with AlexNet, GoogleNet, and ResNet and the accuracies reported were 99.17, 95.83, and 93.33%, respectively (Tuncer and Alkan, 2022). The time-domain features and pattern recognition networks were used for performance evaluation using EMG signals for the classification of hand gestures where a maximum accuracy of 97.3% was obtained for the finger movement dataset and a maximum accuracy of 98.87% was obtained for the hand grasp dataset (Vasanthi and Jayasree, 2020). The multiclass myoelectric identification of five-finger motion using an ANN with a classification accuracy of 98.7% and using a SVM with a classification accuracy of 96.7% was reported in Ahmad et al. (2017). The performance analysis of classifiers for EMG signals of various hand movements in LABVIEW software was reported in Dev and Singh (2016). The EMG signal classification of wide range motion signals for prosthetic hand control was done with a K-nearest neighbour classifier reporting a high classification accuracy of 98.9% (Mahmood et al., 2021).

The main contributions of this work are as follows:

- Once the basic pre-processing of sEMG signals is done by using a simple independent component analysis (ICA) technique, the proposed methodologies are then implemented. The first technique proposed is a dynamic graph construction and graph entropy-based classification of sEMG signals for finger movement classification.
- The second technique proposed encompasses the ideas of dimensionality reduction, evolutionary algorithms (EA), Bayesian belief networks (BBN) and extreme learning machines (ELM) for the classification of sEMG signals, from which a hybrid technique called as EA-BBN-ELM is developed.
- The third technique proposed utilizes the ideas of differential entropy (DE), fuzzy cognitive maps (FCM), and empirical wavelet transformation (EWT), and a hybrid model called DE-FCM-EWT with machine learning classifiers was developed for the classification of sEMG signals.
- The fourth technique proposed uses the ideas of local mean decomposition (LMD) and fuzzy C-means clustering along with a combined kernel least squares support sector machine (LS-SVM) classifier.

The organization of the research is as follows: Section 2 describes each of the proposed methodologies in detail, and is followed by the results and discussion in Section 3 and conclusion in Section 4.

## 2. Proposed strategies

The proposed strategies are explained in detail in the following subsections.

### 2.1. Proposed strategy 1: dynamic graph construction and graph entropy-based classification

#### 2.1.1. Construction of dynamic graph

**Figure 1:**
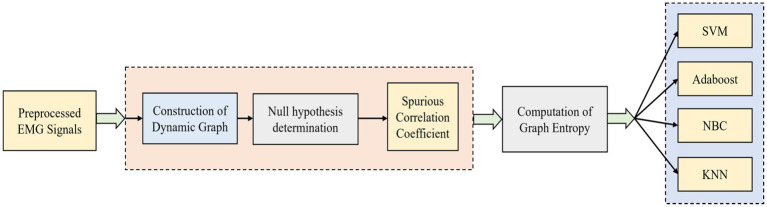
Simplified illustration of dynamic graph construction and graph entropy-based classification.

Among a multiple time series, causality is initially identified so that the spurious correlation coefficient can be easily computed (Goyal et al., 2020). For the particular time series p and q , the causality between them is represented as:

(1) $$C\left(p,q\right)=\{\begin{array}{c} 1\;\;\begin{array}{cc} if & \begin{array}{cc} prob<0.05 & \end{array} \end{array}\\ 0\begin{array}{ccc} & & \begin{array}{ccc} otherwise & & \end{array} \end{array} \end{array}$$

where the causality between the time series p and q is indicated by C(p,q) . The probability of showing that the two series are not causally related is expressed by the probability ‘prob.’ With the help of the Granger causality test, the computation of the causality between the time series p and q can be done. A null hypothesis is made considering that there is no causal relationship between the two-time series. To predict the time series q , the Granger causality test is proposed so that two regressions can be utilized. The past values of the series q are used to predict the current value of q by the first regression. The past values of p and q are used to predict the current value of q by the second regression. If the first prediction is outperformed by the second prediction, then it specifies that the prediction performance for the time series q is improved by the past values of time series p . For these two regressions, the sum squared residuals (SSR) can be easily computed. To test the null hypothesis, the *t*-test and *F*-test is utilized by the Granger causality test by means of utilizing the SSR of two regressions. These are done to check whether the prediction of the series q is improved significantly by the series p . If the null hypothesis is true, then the ‘prob’ value is the probability of observing a particular data more extreme than the current one. If the ‘prob’ value is small, it implies that the two series exhibit causality as there is only a small probability. The null hypothesis can be easily rejected based on the principle of small probability. In hypothesis testing, the concept of significance is quite important as it indicates the probability of rejecting the null hypothesis when it is true. In our experiment, the significance level is assigned to 0.05. Between the two series, the probability of the causality is less than 5% if the probability value is less than 0.05. The null hypothesis can be rejected in such a case and so we could say that a causal relationship exists between the time series p and q . By means of using the Pearson correlation coefficient (PCC) and the causality, the computation of the spurious correlation coefficient is done and is formulated as:

(2) $$R\left(p,q\right)=\{\begin{array}{c} 1\begin{array}{cc} & if\begin{array}{cc} & C\left(p,q\right)=0 \end{array} \end{array}\\ C\left(p,q\right)-\left|PCC\right|\begin{array}{cc} & otherwise \end{array} \end{array}$$

where the causality between the time series p and q is mentioned by C(p,q) . The spurious correlation coefficient is indicated by R(p,q) . When the two correlated time series are not causally related, then the conception of the spurious relationship takes place. Therefore, a spurious correlation shares an inversely proportional relationship with the causality between the time series. The spurious correlation coefficient R(p,q) is 1 when the causality of C(p,q) is 0. The spurious correlation coefficient is large if the probability of causality between the series p and q is small. With the help of the spurious correlation coefficient, a graph can be easily built. For a graph G=(V,E) , the set of the vertices in the graph is indicated by V and the set of edges in the graph is indicated by E . The channel is specified by the vertices for the multi-channel EMG signals. Between the two channels of the EMG signals, the spurious correlation coefficient is indicated by the weight of the edges (Goyal et al., 2020). The dynamic graph is indicated as $G=\left\{G_{t\to }G_{t+1}|G_{i}\in G,t\in \left[0,T\right]\right\}$ , where T represents the total number of time intervals, $G_{i}$ indicates the graph at time interval i , and $G_{t}$ specifies the precedent graph of $G_{t+1}$ . Figure 1 shows a simplified illustration of the dynamic graph construction and graph entropy-based classification.

#### 2.1.2. Computation of graph entropy

To assess the similarity between the two graphs, the graph entropy is used (Dehmer, 2008). For a graph G=(V,E) , the computation of the entropy of the vertex $v_{i}$ is done utilizing the weights of the edges which are connected to it. If there is a connection between vertex $v_{i}$ and vertex $v_{j}$ , the formulation of the entropy of the vertex $v_{i}$ is calculated as follows:

(3) $$e\left(v_{i}\right)=-\underset{j=0,j\neq i^{N}}{\sum }R\left(v_{i},v_{j}\right)\log R\left(v_{i},v_{j}\right)$$

where the total number of vertices connected with the vertex $v_{i}$ is denoted as N . The spurious correlation coefficient between the vertices $v_{i}$ and $v_{j}$ is expressed as $R\left(v_{i},v_{j}\right)$ .

To assess the similarity between the two graphs, the exploitation of graph entropy is computed by utilizing the entropy of the vertices and is expressed as:

(4) $$e\left(G\right)=\underset{i=0^{N}}{\sum }e\left(V_{i}\right)$$

where the number of vertices is represented by N and the entropy of the vertex $v_{i}$ is specified by $e\left(v_{i}\right)$ . The formulation of the entropy from the graph in the particular time interval t∈[0,T] is expressed as:

(5) $$\varepsilon =\left\{e\left(G_{t}\right)|t\in \left[0,T\right]\right\}$$

Once the graph entropy features are obtained, they are then fed into the machine learning classifiers to compute the output.

### 2.2. Proposed strategy 2: dimensionality reduction with EA-based BBN-ELM

The expression of the sEMG dataset is initially mentioned here in a n×D matrix P which has m data vectors $p_{i}\left(i\in \left\{1,2,\ldots ,n\right\}\right)$ with a specific dimensionality D . In this dataset, an intrinsic dimensionality ${}^{'}d^{\prime }$ is possessed (where d<<D ). The transformation of the dataset P with a dimensionality D into a new dataset Q with dimensionality d is done with the aid of dimensionality reduction techniques, while the geometry of the data is hold on firmly. The dimensionality of the sEMG dataset is reduced with the help of local tangent space alignment (LTSA) and local linear coordination (LLC) and then the EA-BBN-ELM model is implemented to compute the output.

#### 2.2.1. LTSA

The local tangent space of all datapoints describing the native possessions of the high dimensional data is utilized by the LTSA (Huo and Smith, 2009). Linear mapping is present from a high dimensional datapoint to its local tangent space if there is conjecture of the local linearity and the manifold. Similarly, from the corresponding low-dimensional datapoint, linear mapping is again extended to the same local tangent space. Therefore, from the low-dimensional representation, the local tangent space of the manifold is constructed and therefore the linear mapping is aligned in a proper way by LTSA. The co-ordinates of the lower-dimensional data representations are searched completely by the LTSA. Also, the searching of the linear mapping of the low dimensional data points is done to the high dimensional data’s local tangent space. At the datapoint $p_{i}$ , the local tangent spaces are computed by LTSA. For the k datapoints $p_{i_{j}}$ , Principal Component Analysis (PCA) is implemented as they are neighbors of datapoints $p_{i}$ . Therefore, the neighborhood of $p_{i}$ is mapped through mapping $M_{i}$ to the local tangent space $\Theta_{i}$ . Linear map $L_{i}$ is present from the local tangent space co-ordinates $\theta_{i_{j}}$ to the low-dimensional representations $q_{i_{j}}$ . The minimization problem expressed below is performed by LTSA by means of utilizing the possession of the local tangent space as:


(6) $$\underset{Q_{i},L_{i}}{\min}\underset{i}{\sum }∥Q_{i}C_{k}-L_{i}\Theta_{i}{∥}^{2}$$

where the centering matrix of size k is represented as $C_{k}$ . The alignment matrix A is from the eigen vectors and so the minimization solution can be found corresponding to the d non-zero and smallest values of A . By means of iterative summation, the alignment matrix entries A are obtained as:


(7) $$A_{s_{i}s_{i}}=A_{s_{i}s_{i}}+C_{k}\left(I-V_{i}V_{i}{}^{T}\right)C_{k}$$

where the selection matrix is expressed as $S_{i}$ containing the indices of the nearest neighbor of datapoints $p_{i}$ . By means of computing the eigen vectors which match to the ${}^{'}d^{\prime }$ non-zero and smallest eigen vectors of the matrix $\frac{1}{2}\left(A+A^{T}\right)$ , the low-dimensional representation Q is obtained.

#### 2.2.2. LLC

A number of locally linear models are computed initially and then a global arrangement of the linear models is done in a subsequent manner. There are two important steps in LLC: initially, using an expectation–maximization (EM) algorithm, the mixture of local linear models is computed and, secondly, using a variant of LLE, the low-dimensional data rendition is obtained by means of aligning the local linear models (Roweis et al., 2001). Using the EM algorithm, a mixture of m factor analyzers are initially constructed by LLC. The engagement of mixture probabilistic PCA models can also be done. The construction of m data representations $y_{ij}$ and their respective responsibilities $r_{ij}$ is utilized by the local linear models in the mixture for every datapoint $p_{i}$ . The datapoint $p_{i}$ corresponding to the mode j is described by the responsibilities $r_{ij}$ , so that $\underset{j}{\sum }r_{ij}=1$ is satisfied. The computation of the responsibility weighted data representations $u_{ij}=r_{ij}y_{ij}$ are done utilizing the local models and its respective responsibilities. In a n×mD block matrix U , the storage of the responsibly weighted data representation, $v_{ij}$ are done. Depending on U and on a matrix M , the performance of the local models’ alignment is done and is expressed as:


(8) $$M={\left(I-W\right)}^{T}\left(1-W\right)$$

LLE computes the reconstruction weights for the matrix W and the n×n identity matrix is indicated by I . By means of solving the generalized eigen problem, the local models are aligned by LLC and represented as:


(9) $$Be_{v}=\lambda Ae_{v}$$

for the d non-zero and smallest eigen values. B represents the in product of $M^{T}U$ and A represents the in product of U . Thus, the matrix L is found by the d eigen vectors $e_{v_{i}}$ and is represented as a linear depiction from the data representation U which is responsibly weighted to the data representation with the underlying low dimension Q . By means of computing Q=UL , the low dimensional data representation is easily obtained. Once the low dimensional data representation is obtained, it is then fed into the EA-BBN-ELM model.

#### 2.2.3. Bayesian belief networks

**Figure 2:**
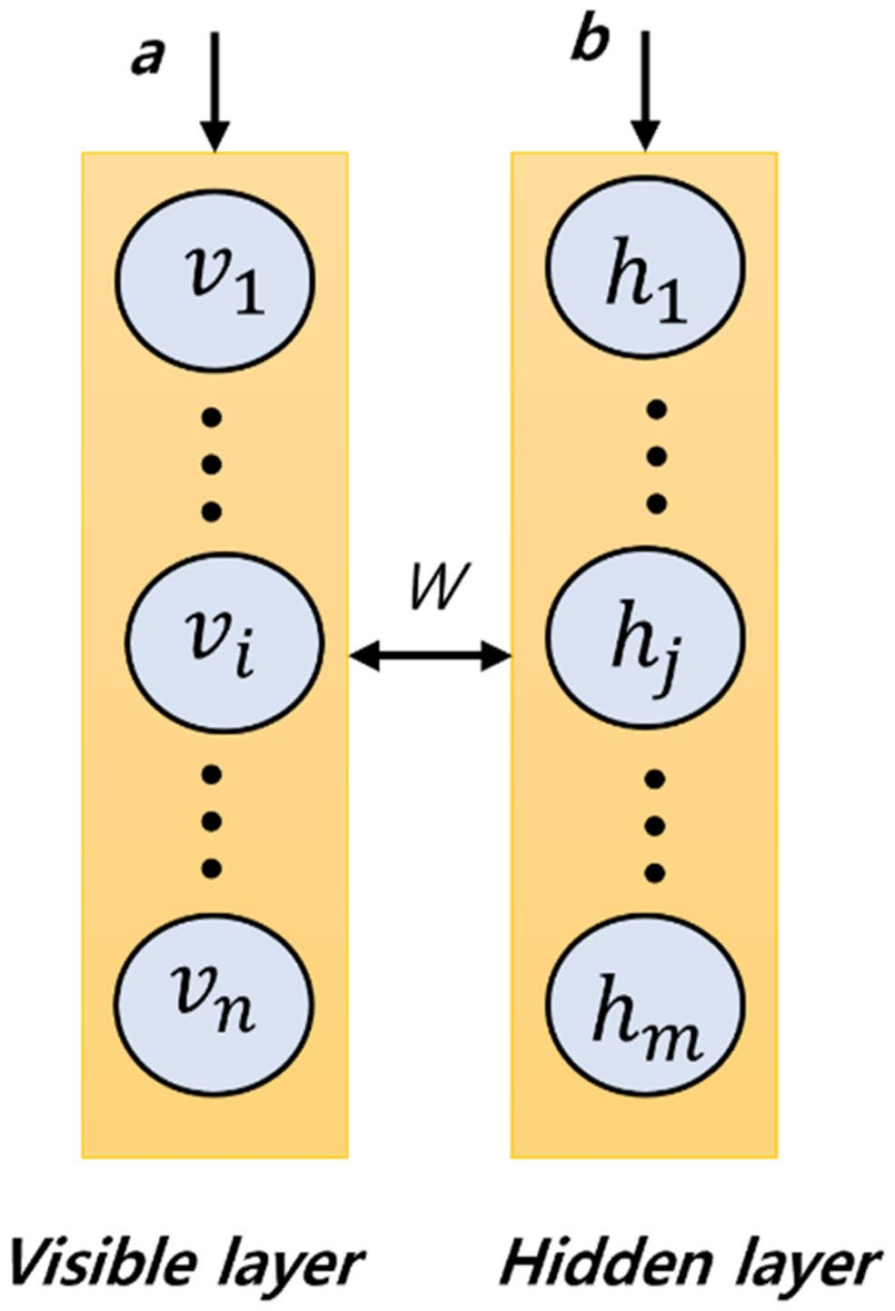
Illustration of a restricted Boltzmann machine.

A famous type of deep neural network that utilizes restricted Boltzmann machines (RBM) as learning models is the Bayesian Belief Network (BBN; Cheng et al., 1997). An RBM is a generative stochastic neural network that can learn the probabilistic distribution of its inputs without supervision. A set of visible units are present in an RBM and represented as $v\in {\left\{0,1\right\}}^{n}$ , and a set of hidden units are present in an RBM and represented as $h\in {\left\{0,1\right\}}^{m}$ , where n is the number of visible units and m is the number of hidden units. With the help of an asymmetrically weighted connections matrix (W) , the connection of the two layers is done and, in between the neurons within a layer, there are no connections. The structure of an RBM with n visible units and m hidden units is shown in the Figure 2, where a and b represent the biases of visible and hidden layers. The stacking of RBMs can be done in an end-to-end manner and can be trained greedily so that a BBN can be formed. The main idea behind a BBN is that the learning rule for updating weight using RBM is captured as follows:

(10) $$\Delta w_{ij}=\varepsilon {\left(v_{i}h_{j}{}_{data}-v_{i}h_{j}{}_{\mathrm{mod}el}\right)}_{t}$$

where $.\ldots_{d}$ indicates the expectation of a D distribution and the learning rate is expressed as ε . For the updating of bias parameters, the rules are set as follows:

(11) $$\Delta a_{i}=\varepsilon \left(v_{i}{}_{data}-v_{i}{}_{\mathrm{mod}el}\right)$$

(12) $$\Delta b_{j}=\varepsilon \left(h_{j}{}_{data}-h_{j}{}_{\mathrm{mod}el}\right)$$

The RBM is a biographic graph as the computation of $v_{i}h_{j}{}_{data}$ is quite simple. Depending on the hidden node, the activations of a visible node can be made mutually independent and is expressed as follows:

(13) $$P\left(v|h\right)=\overset{n}{\underset{i=1}{\prod }}P\left(v_{i}|h\right)$$

Depending on the hidden vector, the mood of a visible vector is expressed as follows:

(14) $$P\left(v_{i}=1|h\right)=\delta \left(b_{i}+\Sigma_{j}W_{ij}h_{j}\right)$$

where the logistic sigmoid function is expressed as δ , and it is defined as δ(x)=1/(1+exp(−x)) .

The binary state $h_{j}$ of hidden unit j is adjusted to 1 for the randomly selected training input v with a particular probability depending on the following equation:

(15) $$P\left(h_{j}=1|v\right)=\delta \left(c_{j}+\Sigma_{i}W_{ij}v_{i}\right)$$

The hidden unit is turned on if the random number value is in the interval (0,1) and with a uniform distribution. For the computation of the second part of Equation 10, a plethora of algorithms have been utilized. The standard algorithm proposed is contrastive divergence with one step of Gibbs sampling (CD-1). The visible units $v_{i}$ are initialized to the input distance to compute the $v_{i}h_{j}{}_{\mathrm{mod}el}$ in CD-1. Then the moods of the hidden units $h_{j}$ are computed and finally $v_{i}^{'}$ and $h_{j}^{'}$ are computed using a one-step reconstruction of v and h nodes. Finally, the updating of the weight and biases are done as follows:

(16) $$\Delta W_{ij}=\varepsilon \left(v_{i}h_{j}-datav_{i}^{'}h_{j}^{'}{}_{reconstruction}\right)$$

(17) $$\Delta b_{i}=\varepsilon \left(v_{i}-datav_{i}^{'}{}_{reconstruction}\right)$$

(18) $$\Delta c_{j}=\varepsilon \left(h_{j}-datah_{j}^{'}{}_{reconstruction}\right)$$

There are three important steps in BBN learning algorithms. To initialize the network, an unsupervised pre-training step via RBMs is done. Connection weights are assigned between the RBM hidden nodes and the output node by fine-tuning. Backpropagation (BP) is used so that the network weights are allowed to be refined layer by layer. The network weights are adjusted by BP so that the training samples T are indicated by the output neurons O . The difference between expected output T and the actual output O is a squared error and is computed as:

(19) $$Error={\left(T-O\right)}^{2}$$

#### 2.2.4. Extreme learning machine

An efficient learning algorithm of the single hidden layer feed forward neural networks (SLFN) family is the ELM (Castaño et al., 2013). In between the input nodes and hidden nodes, the connection weights are initialized randomly in ELM. The random initialization also takes place in the hidden node biases and the least squares approach is used to compute the connection weights between the hidden nodes and the output node. For a binary classification problem, the ELM classification function is expressed as:

(20) $$f\left(x\right)=\delta \left(\overset{m}{\underset{i=1}{\sum }}\beta_{i}h_{i}\left(x\right)\right)=\delta \left(h\left(x\right)\beta \right)$$

where the connection weight vector between the hidden layer and output node is represented as $\beta ={\left[\beta_{1},\ldots ,\beta_{m}\right]}^{T}$ . For multi-class classification problems, the classification function must be used accordingly (Castaño et al., 2013). The output vector from the hidden layer is represented as $h\left(x\right)=\left[h_{1}\left(x\right),\ldots ,h_{m}\left(x\right)\right]$ according to input x . The mapping of the data is done by h(x) from the d-dimensional input space to the m-dimensional feature space of hidden layer H . An ELM has the lowest norm of β in addition to having the smallest learning error and so a higher network classification performance is achieved.


(21) $$\mathrm{Minimize:}H\beta -T^{2},\beta$$

where the output matrix of hidden nodes is represented as H and shown in the following equation.

(22) $$H=\left[\begin{array}{c} h\left(x_{1}\right)\\ :\\ h\left(x_{N}\right) \end{array}\right]=\left[\begin{array}{ccc} h_{1}\left(x_{1}\right) & \ldots & h_{m}\left(x_{1}\right)\\ : & : & :\\ h_{1}\left(x_{N}\right) & \ldots & h_{m}\left(x_{N}\right) \end{array}\right]$$

**Figure 3:**
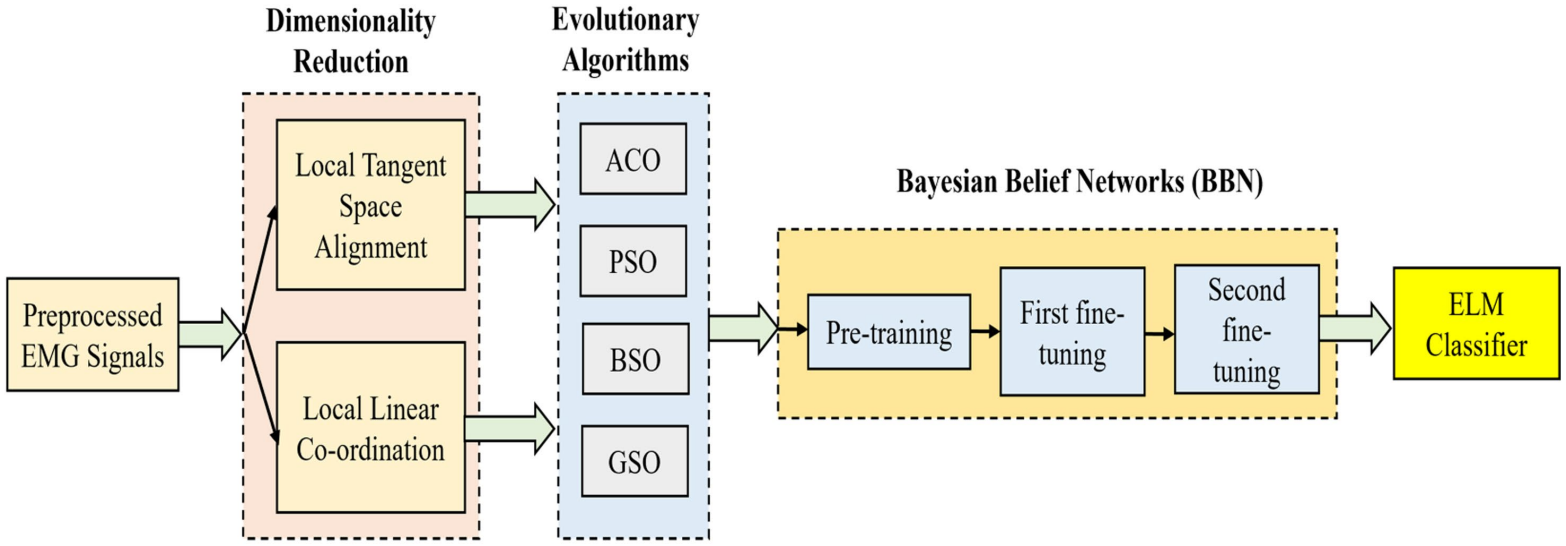
Simplified illustration of the proposed EA-BBN-ELM model technique.

The output weights vector β can be computed easily using the following equation if matrix $T={\left[y_{1},\ldots ,y_{N}\right]}^{T}$ is computed with sample labels as follows:

(23) $$\beta =H^{†}T=H^{T}{\left(HH^{T}\right)}^{-1}T$$

where the Moore-Penrose generalized inverse of matrix H is represented as $H^{†}$ . The overall block diagram of this proposed technique is illustrated in Figure 3.

#### 2.2.5. Ensemble learning model of EA-BBN-ELM

To learn the relationships between the input and output data, ANNs are quite useful and versatile. They are quite powerful when utilized for a variety of applications such as classifications, predictions, clustering, and control systems. The most used training algorithm for NN is a BP feed-forward network. At the start of the network training process, random connection weights are used and are one of the major challenges of using a BP. A better classification performance can be provided by the local search feature in BP. One of the main advantages of BBN pre-training is that more reasonable weights can be used instead of random weights. In between the last hidden nodes and the output nodes, the connection weight matrix is generated randomly by the BBN and the problem can be overcome by using an EA-BBN-ELM hybrid model. Once the pre-training is done using RBMs to the end of the network, the addition of an ELM is done so that the connection weights between the hidden layer and output layer ( β ) matrix is computed. Therefore, the matrix W of the ELM is equivalent to the weights matrix considered from the final RBM of the BBN and the computation of matrix β is done. The calculation of the error is done and then the implementation of the BP algorithm happens so that the network weights are updated. For a BNN, searching the proper topology is a search issue, where the main goal is to trace the optimal topology for the network. To address this challenge, standard evolutionary algorithms (EA) like ant colony optimization (ACO)/particle swarm optimization (PSO)/backtracking search optimization (BSO)/glowworm swarm optimization (GSO) techniques etc. are used to find the optimal or near optimal solution in various types of objective functions (Zhang et al., 2015). An EA-BBN-ELM network as a whole improves the BBN learning and can help in the optimization of network topology using EA also.

### 2.3. Proposed strategy 3: DE-HFCM-EWT hybrid model with classifiers

#### 2.3.1. Differential entropy

In the continuous probability distribution, the degree of uncertainty is quantified by the DE and is a continuous form of Shannon entropy (Wu, 1996). If a continuous variable x is assumed along with its probability density function p(x) , then the differential entropy when the variable x obeys the Gaussian distribution $N\left(\mu ,\sigma^{2}\right)$ is expressed as:

(24) $$\begin{array}{l} h\left(x\right)=-\overset{\infty }{\underset{-\infty }{\int }}\frac{1}{\sqrt{2\pi \sigma^{2}}}e^{-\frac{{\left(x-\mu \right)}^{2}}{2\sigma^{2}}}\log\left(\frac{1}{\sqrt{2\pi \sigma^{2}}}e^{-\frac{{\left(x-\mu \right)}^{2}}{2\sigma^{2}}}\right)dx\\ =\frac{1}{2}\log\left(2\pi e\sigma^{2}\right) \end{array}$$

where the constants are represented by π and e . A linear relationship is present between the differential entropy of a Gaussian variable and its respective variance. For $x∼N\left(\mu ,\sigma^{2}\right)$ , the variance is expressed as:

(25) $$\sigma^{2}=\frac{1}{N}\Sigma {\left(x-\mu \right)}^{2}$$

where the number of samples is represented as N . The previous formula is simplified to $\sigma^{2}=\frac{1}{N}\Sigma x^{2}$ when the mean value is zero and it specifies the mean energy of variable x . Thus, by using discrete Fourier transform (DFT), the estimation of the variance of Gaussian variables from the energy can be calculated as follows:

(26) $$h\left(x\right)=\frac{1}{2}\log\left(E\right)+\frac{1}{2}\log\left(\frac{2\pi e}{N}\right)$$

where the energy spectrum of the signal is represented as E . The raw signal may not be assessed following the Gaussian distribution as sEMG recordings are non-stationary signals and so the estimation of the differential entropy cannot be done directly; therefore, with the implementation of FCM, this issue can be easily solved.

#### 2.3.2. Fuzzy cognitive maps (FCM)

FCMs are simply weighted directed graphs where the concepts are represented by nodes and the logical relations are represented by edges (Kosko, 1986). For a FCM which has $N_{c}$ nodes, the concept state values are defined as a vector S , $S=\left(S_{1},S_{2},\ldots ,S_{N_{c}}\right)$ , where $S_{i}\in \left[0,1\right]$ or [−1,1], $i=1,2,\ldots ,N_{c}$ .

The activations value of node i is represented by the state value $S_{i}$ . With the help of a $N_{c}\times N_{c}$ matrix M , the representation of the logical relationships among the different nodes are done as follows:

(27) $$M=\left[\begin{array}{cccc} m_{11} & m_{12} & .. & m_{1N_{c}}\\ m_{21} & m_{22} & .. & m_{2N_{c}}\\ : & : & .. & :\\ m_{N_{c}1} & m_{N_{c}2} & .. & m_{N_{c}N_{c}} \end{array}\right]$$

where $m_{ij}\in \left[-1,1\right]$ indicates the strength of node $j^{\prime }$ s impact on node i . The negative $m_{ij}=-\left|a\right|$ indicates that a negative impact on node i is represented by node j with a strength |a| . The $m_{ij}=0$ indicates that there is logical relationship between node i and j . The node j has a positive impact on node i and that is represented by the positive $m_{ij}=\left|a\right|$ with a strength |a| . The weight matrix M influences the state value of a node at $t+1^{th}$ iteration and it has an equal influence on all the state value of connected nodes at $t^{th}$ iteration. The dynamics of the FCM can be expressed by the following equation as follows:

(28) $$S_{i}\left(t+1\right)=g\left(\overset{N_{c}}{\underset{j=1}{\sum }}m_{ij}S_{j}\left(t\right)\right)$$

where the state value of node j at $t^{th}$ iteration is indicated by $S_{j}\left(t\right)$ and the non-linear trace function is indicated by g() . For FCMs, a lot of transformation functions are available. A hyperbolic tangent function is utilized to locate the state values in the range [−1,1] and is defined as follows:

(29) $$\tanh\left(x\right)=\frac{e^{x}+e^{-x}}{e^{x}-e^{-x}}$$

Short term temporal relationships can be easily modeled by FCMs based on Equation 28. Therefore, to model the long temporal dependencies, the high-order FCMs (HFCMs) are utilized. The modeling process of an h order HFCM is specified as:

(30) $$S_{i}\left(t+1\right)=g\left(\begin{array}{l} \overset{N_{c}}{\underset{j=1}{\sum }}m_{ij}^{1}S_{j}\left(t\right)+m_{ij}^{2}S_{j}\left(t-1\right)+.\ldots +\\ m_{ij}^{h}S_{j}\left(t-h-1\right)+m_{i0} \end{array}\right)$$

where the bias term is expressed as $m_{i0}$ and at time step t−h+1 the strength of node $j^{\prime }s$ impact on node i is represented as $m_{ij}^{h}$ .

#### 2.3.3. EWT

**Figure 4:**
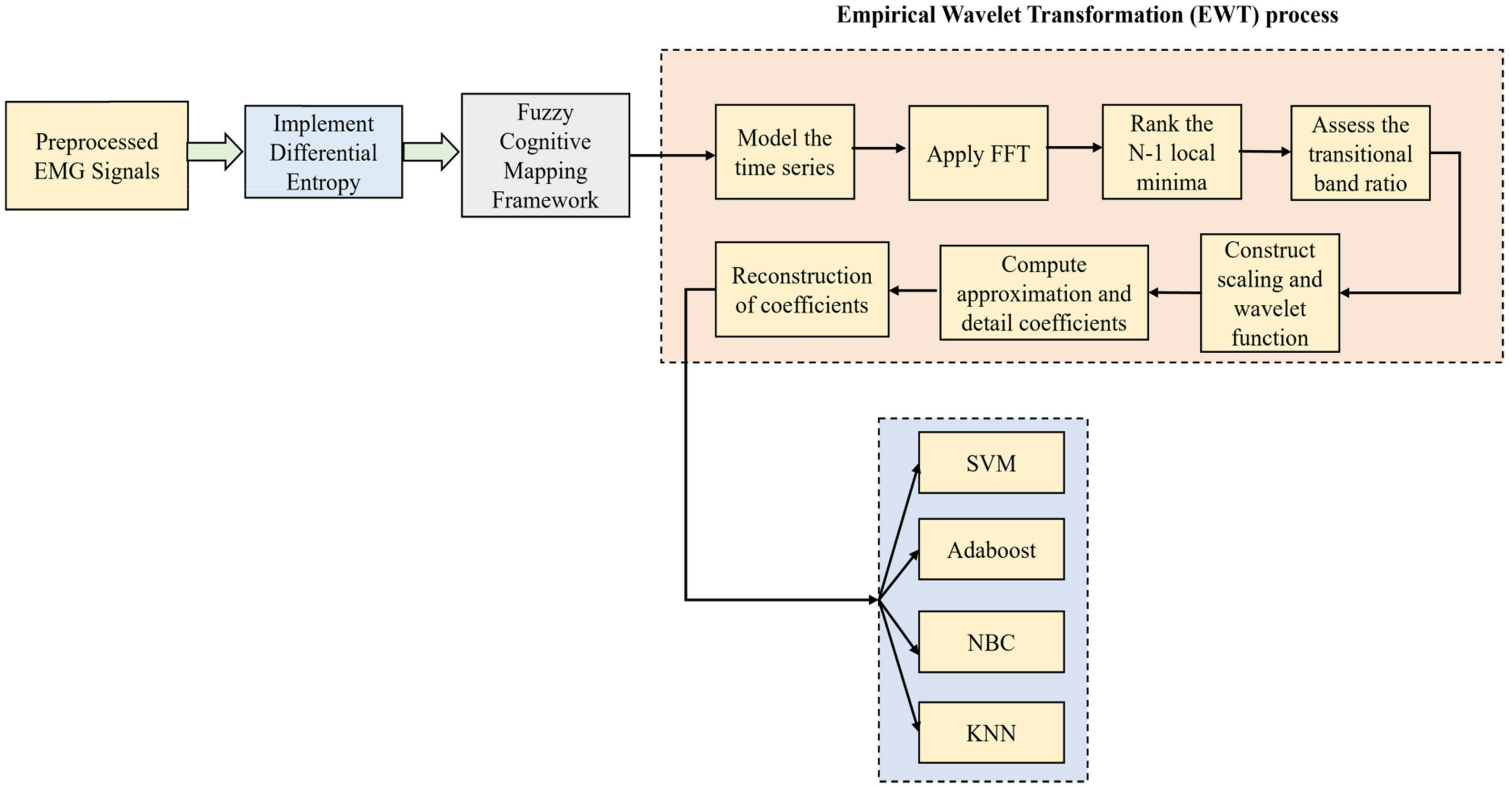
Overall framework of the DE-based FCM and EWT with machine learning classifiers.

The FCM-modeled network is now computed with EWT as it is one of the famous data-driven adaptive signal decomposition techniques used (Gilles, 2013). For the analysis of non-stationary time series data, it has an effective performance along with an established theoretical foundation. It has been used widely in time series modeling and signal processing applications. The signal is analyzed directly in the Fourier domain by EWT after the Fast Fourier Transform (FFT). Then the spectrum separation is implemented through band-pass filtering with the aid of a specific filter bank. Figure 4 shows the overall framework of the DE-based FCM and EWT with machine learning classifiers.

The proposed algorithm steps for the novel DE-HFCM-EWT are explained as follows:

1. The padding of the time series f(t) is done initially with the help of K-nearest neighbors (KNN) algorithm so that the boundary effect in EWT can be prevented.
2. The differential entropy is computed.
3. All the subseries $S_{i}\left(t\right)$ are generated by implementing EWT to the training set of f(t) by means of including all the padded data points.
4. The discrete version of its spectrum F(w) is obtained by means of performing FFT to f(t).
5. The corresponding frequency of ranked local minima are determined in the spectrum.
6. To assess the empirical wavelet, the transitional band ratio γ is defined.
7. The scaling and wavelet functions are well established.
8. The approximate coefficients of the signals are computed in detail.
9. The structure of HFCM is identified in detail and the weights optimized.
10. The trained model is applied along with the test model to the machine learning classifiers.

### 2.4. Proposed strategy 4: LMD-based fuzzy C-means clustering and LS-SVM classifier

#### 2.4.1. Local mean decomposition

A non-linear signal analysis used generally is LMD (Wang et al., 2018). The sEMG signal is decomposed adaptively into a sum of series of functional components so that the intrinsic nature of the signals can be reflected well. The non-stationarity of the signal can be reduced well by the ensemble decomposition methods. The commonly used ensemble decomposition models are LMD, empirical mode decomposition (EMD), ensemble EMD (EEMD), and complementary EEMD (CEEMD). Some unique advantages are present in LMD as the decomposition of the signal can be done adaptively based on its own characteristics. After decomposition using LMD, every component has some physical significance and can easily reflect the inherent nature of the signal. The end effect too can be well restrained with the help of LMD so that the integrity of signal information is preserved and the calculation time can be reduced well. For any original sEMG signal x(t) , the decomposition of the LMD algorithm is a process of multiple cycles. From the original signal, the pure frequency modulation signal and envelope signal can be extracted by using the LMD algorithm. By multiplying these two kinds of signals, a functional component can be easily obtained. By gradually cycling, all the functional components can be easily obtained and thus the instantaneous frequency and amplitude too can be ultimately obtained. From the pure frequency modulated signal, the instantaneous frequency can be easily obtained. The amplitude modulation information and the frequency modulation information of the functional components are represented by the instantaneous frequency. Ultimately, for the full original signal, the entire time-frequency distribution is obtained. x(t) is the original signal and the intermediate variables are represented as h(t) and n(t) . The envelope function is represented as $a_{i}\left(t\right)$ and the pure frequency component is represented by $S_{i}\left(t\right)$ . The vital function component is expressed by $F_{i}\left(t\right)$ , the local extremum point is expressed by $n_{i}\left(t\right)$ , and the local mean function is expressed by $m_{i}\left(t\right)$ . The decomposition of the original sEMG signal x(t) is done into a K functional component and a new residual signal $r_{k}\left(t\right)$ by LMD algorithm as follows:

(31) $$x\left(t\right)=\overset{K}{\underset{n=1}{\sum }}F_{n}\left(t\right)+r_{k}\left(t\right)$$

**Figure 5:**
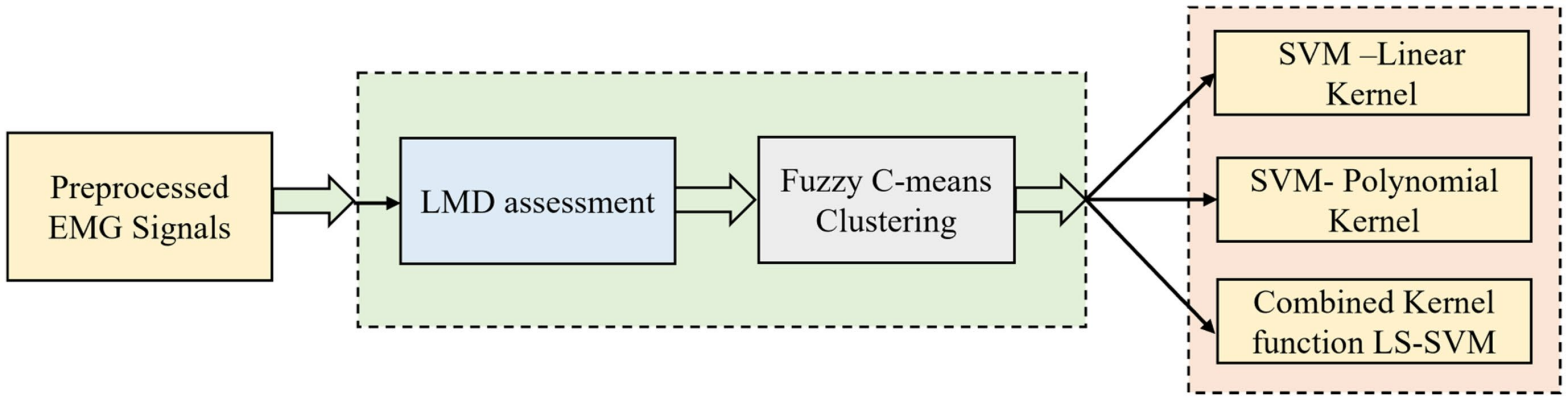
Simplified illustration of LMD-fuzzy C-means combined kernel function LS-SVM.

The simplified illustration of this proposed method is shown in Figure 5.

#### 2.4.2. Fuzzy C-means clustering

The fuzzy C-means algorithm is a very popular distance-based portioning clustering algorithm (Krishnapuram and Keller, 1993). The clustering partition is performed iteratively until its target reaches a minimum. A membership matrix is used by the fuzzy C-means algorithm so that each sample is divided based on its probability of belonging to each category. To multiple categories, the samples can belong and the probability of belonging to every such category is different. The fuzzy C-means algorithm is actually a statistical technique and the division process is actively the method of optimization of the target formula. The data object set $P=\left\{p_{1},p_{2},\ldots ,p_{n}\right\}\subset {ℜ}^{d}$ is a limited collection of data objects and element $p_{i}$ is a d-dimensional vector. The membership matrix $U_{ij}$ is found by the fuzzy C-means algorithm so that the degree of each data element pertaining to each cluster is denoted by it. The following objective function is optimized by the partition process as follows:

(32) $$J_{m}=\overset{N}{\underset{i=1}{\sum }}\overset{C}{\underset{j=1}{\sum }}u_{ij}^{m}p_{i}-c_{j}{}^{2}$$

where the weighting exponent is expressed as m and it is called the fuzzy weighting coefficient. The fuzzy weighting coefficient can be any real number greater than 1. The degree of $p_{i}$ in the cluster $c_{j}$ is expressed as $u_{ij}$ . The $i^{th}$ input data is represented as $p_{i}$ and the center of the $j^{th}$ cluster is represented as $c_{j}$ . The computation of the cluster center $c_{i}$ is done as follows:

(33) $$c_{i}=\frac{\sum_{j=1}^{n}u_{ij}^{m}p_{j}}{\sum_{j=1}^{n}u_{ij}^{m}}$$

The membership matrix is computed as follows:

(34) uij=1∑k=1c(dijdkj)2(m−1)

The basic steps of the fuzzy C-means algorithm are as follows:

(1) The membership matrix U is initialized with a random number between 0 and 1 so that the constraint is satisfied.
(2) The clustering centroids $c_{i}$ are computed, i=1,2,…,c
(3) The value function is also computed. If the obtained result is less than a defined threshold value, then the algorithm terminates.
(4) The new membership matrix U is computed and then return to Step 2. The fuzzy clustered feature values after the assessment of LMD methodology are directly fed into the combined kernel function LS-SVM.

#### 2.4.3. Combined kernel function LS-SVM

The mapping of the sample space of the LSSVM is done into a high-dimensional feature space through the non-linear mapping function. For a sample dataset $\left\{p_{t},q_{t}\right\}$ , t=1,2,…,N , there is:

(35) q=wφ(p)+b

where the total number of samples is represented as N ; $q,q\in {ℜ}^{d}$ is the output of the non-linear systems; $p,p\in {ℜ}^{m}$ is the input of non-linear systems. The dimension of sample space is represented by m . The mapping function of input space with m -dimensional to output space with high d -dimensional (d≫m) is represented as φ() . The weight coefficient vector is represented as $w,w\in {ℜ}^{d}$ and the constant bias is represented as b . By minimizing the next objective function, the optimal w and b can be obtained as follows:

(36) $$\underset{w.b,e}{\min}J\left(w,e\right)=\frac{1}{2}w^{T}w+\frac{1}{2}\gamma \overset{N}{\underset{t=1}{\sum }}e_{t}^{2}$$

where the structural form is represented by J(w,e) . The permissible error is represented by $e_{t},t=1,2,\ldots ,N$ and it implies that there is a prediction error between the original and predictive output. To control the degree of penalty, the regularization parameter used is γ . To solve such a constrained optimization problem, the Lagrange function is used as follows:

(37) $$L\left(w,b,e,a\right)=J\left(w,e\right)-\overset{N}{\underset{t=1}{\sum }}a_{t}\left(w^{T}\varphi \left(p_{t}\right)+b+e_{t}-q_{t}\right)$$

where the Lagrange multiplier is represented by $a_{t}$ . The kernel function K() and the mapping function φ() should satisfy the following equation as follows:

(38) $$K\left(p_{t},p_{j}\right)=\varphi {\left(p_{t}\right)}^{T}\varphi \left(p_{j}\right)$$

The kernel function is used to assess the non-linear mapping ability of the LS-SVM prediction model (Maria et al., 2014). To map the samples from an input space to feature space, the kernel function is used. Generalization ability and learning ability differ for each kernel function. Generally, partial kernel and global kernel functions are the important types of kernel functions. The kernel value will be large if it is farther from the test point if the model uses global kernel functions. The kernel value will be small if it is closer to the test point, if the model uses partial kernel functions. A typical example of a partial kernel function is the radial basis function (RBF) and a typical example of a global kernel function is the polynomial kernel function. Equations 39 and 40 show the polynomial kernel function and RBF kernel function, respectively.

(39) $$K\left(p_{t},p_{j}\right)={\left[\left(p_{t}.p_{j}\right)+1\right]}^{O}$$

(40) $$K\left(p_{t},p_{j}\right)=\exp\left(-\frac{p_{t}-p_{j}{}^{2}}{2\sigma^{2}}\right)$$

The order of polynomial function is represented by O and the width of the RBF kernel function is represented as $\sigma^{2}$ . For the LS-SVM model, the prediction model is represented as:

(41) $$q=\overset{T}{\underset{t=1}{\sum }}\alpha_{t}K\left(p_{t},p_{j}\right)+b$$

In the high dimensional feature space, the complex calculation is avoided by the introduction of the kernel function. One of the vital issues of the LS-SVM is the apt choice of the kernel function (Adankon et al., 2011). A high influence is present on the data points in areas close to the test point by the partial kernel function and therefore it has a weak generalization ability and strong learning ability. On the contrary, the global kernel function has a strong generalization ability and weak learning performance. Thus, the inherent advantages of these two kinds of kernel functions are used to construct the mixed kernel functions.

A good generalization ability is present for the polynomial kernel function. The output of the function will be influenced by the distance of the sample point from the test point. The width of the kernel function is determined by the parameters $\sigma^{2}$ of the RBF. To assess the output of the function, the sample points close to the test point have a major influencing factor and so a good interpolation ability is achieved by the RBF function. Therefore, to have a combined kernel function, the hybrid of these two kernel functions are considered so that a better learning ability and generalization ability in the LS-SVM is achieved. The merits of the two kernels functions are jointly considered and then a better regression prediction performance is obtained. The new hybrid kernel function obtained is as follows:

(42) $$K_{hybrid}=aK_{rbf}+\left(1-a\right)K_{poly},a\in \left[0,1\right]$$

The weight coefficient is indicated as a . The polynomial kernel function is specified as $K_{poly}$ and the RBF kernel function is specified as $K_{rbf}$ . The Mercer condition is satisfied by the $K_{poly}$ and $K_{rbf}$ , so the $K_{hybrid}$ is also used to satisfy the Mercer condition. The proportion of a single kernel to a mixed kernel is assessed by the weight coefficient a . The domination of polynomial kernel function happens if a is greater than 0.5 and the domination of the RBF kernel function happens if a is less than 0.5. The two kernel functions are equally important when a =0.5. When the combined kernel function is constructed, a can be adjusted and the combination of the RBF kernel function and polynomial kernel function can be realized. The partial properties of the RBF kernel function and the global properties of the polynomial kernel function are combined with the help of the combined kernel function to achieve a high generalization ability. Every parameter has a significant impact on the working ability of the LS-SVM if we adopt the combined kernel function. As the parameters are highly interrelated to each other $\left(a,q,\sigma^{2},\gamma \right)$ , the paper employs some metaheuristic algorithms like ACO, GA, and PSO (Zhang et al., 2015) to assess the optimal parameters of the hybrid kernel function LS-SVM prediction model.

## 3. Results and discussion

**Figure 6:**
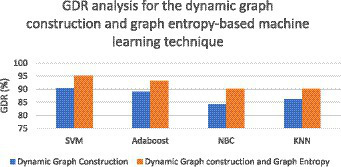
GDR analysis for the dynamic graph construction and graph entropy-based machine learning technique.

**Figure 7:**
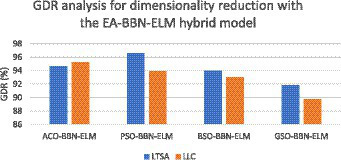
GDR analysis for dimensionality reduction with the EA-BBN-ELM hybrid model.

**Figure 8:**
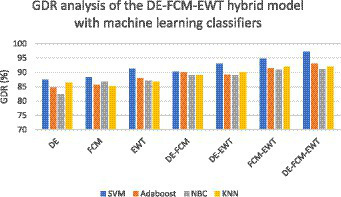
GDR analysis of the DE-FCM-EWT hybrid model with machine learning classifiers.

**Figure 9:**
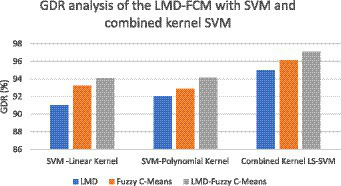
GDR analysis of the LMD-fuzzy C-means with SVM and combined kernel SVM.

The dataset utilized in this paper is from Khushaba and Kodagoda (2012). From eight different participants (six males and two females), the sEMG signals were obtained. The participants were quite healthy and did not report any major neurological disorders. With the help of eight channels, these signals were collected using Delsys sEMG sensors when these participants were made to sit in an armchair. The age range of the participants was between 20 and 35 years and the acquisition of these signals was done at 4,000 Hz. The conversion of these signals was done into a 12-bit format and ultimately 15 movements of the fingers were collected (thumb, index, middle, ring, little, thumb-index, thumb-middle, thumb-ring, thumb-little, hand close, index-middle, middle-ring, ring-little, index-middle-ring, and middle-ring-little), and therefore there are 15 classes in this dataset. There are 24 observations in each class and so there are 360 observations in total (24 × 15 = 360). The analysis was done on this small dataset and the results were analyzed in detail. The standard performance metrics like sensitivity, specificity, and accuracy were computed.

(43) $$Sensitivity=\frac{TP}{TP+FN}$$

(44) $$Specificity=\frac{TN}{TN+FP}$$

(45) $$Accuracy=\frac{TP+TN}{TP+TN+FP+FN}$$

where TP stands for true positive, FP stands for false positive, TN stands for true negative, and FN stands for false negative. As far as the first strategy is concerned regarding the construction of a dynamic graph, the significance value is set to 0.05 only. The experiment was tried with other values as well on a trial-and-error basis but the best results were found only for this value. When the second strategy of the EA-BBN-ELM network was considered, algorithms like ACO, PSO, BSO, and GSO were utilized and the main parameters used were as follows. For ACO, the number of ants was selected as 20, the total number of generations was set as 1,000, the initialization of pheromone was set at 0.8, the weight of pheromone on decision was set as 0.6, the weight of the heuristic data on decision was set at 0.5, and the degree of random choice at random points was assigned a value of 0.1. As far as PSO was concerned, the population size was set at 50, the number of particles was set at 20, the number of iterations at 100, inertia weight at 0.5, and local weights were assigned as 1. For the BSO algorithm, the population size was set at 30 and the mix rate parameter was assigned to 0.5. For the GSO algorithm, the important parameters such as the maximum number of iterations was set at 100, the number of glowworms chosen was 25, the number of neighbors chosen was 10, the constant parameter was set at 0.05, and the step size was assigned at a value of 5. Regarding the third and fourth strategies considered, all the values have been explained in the experimental part itself. Table 1 shows the performance analysis of the dynamic graph construction and graph entropy-based machine learning classifiers. It was observed that a high classification accuracy of 96.44% is obtained when the proposed dynamic graph construction with graph entropy is implemented with the SVM classifier. Table 2 shows the performance analysis of dimensionality reduction with the EA-BBN-ELM hybrid model and the highest classification accuracy of 97.57% is obtained if the PSO-BBN-ELM hybrid model is implemented. Table 3 shows the performance analysis of the DE-FCM-EWT hybrid model with machine learning classifiers and the results show that a high classification accuracy of 98.21% is obtained when classified with the SVM classifier. Table 4 shows the performance analysis of the LMD-fuzzy C-means with combined kernel SVM and the results show that a high classification accuracy of 98.50% is obtained when classified with the combined kernel LS-SVM classifier. The good detection rates (GDR) for the proposed models are also illustrated in Figures 6–9, respectively.

**Table 1:** Performance analysis of dynamic graph construction and graph entropy-based machine learning classifiers.

	SVM			Adaboost			NBC			KNN		
	Sen	Spe	Acc	Sen	Spe	Acc	Sen	Spe	Acc	Sen	Spe	Acc
Dynamic graph construction	92.15	91.15	91.65	90.12	91.12	90.62	86.45	84.59	85.52	88.23	87.23	87.73
Dynamic graph construction and graph entropy	96.56	96.32	96.44	93.34	93.56	93.45	92.23	91.03	91.63	92.34	91.24	91.79

NBC, Naive Bayesian Classifier.

**Table 2:** Performance analysis of dimensionality reduction with the EA-BBN-ELM hybrid model.

	ACO-BBN-ELM			PSO-BBN-ELM			BSO-BBN-ELM			GSO-BBN-ELM		
	Sen	Spe	Acc	Sen	Spe	Acc	Sen	Spe	Acc	Sen	Spe	Acc
LTSA	96.23	95.21	95.72	97.34	97.81	97.57	96.34	95.48	95.91	93.21	92.45	92.83
LLC	96.59	96.08	96.33	95.55	96.23	95.89	95.02	94.98	95	92.76	90.68	91.72

**Table 3:** Performance analysis of the DE-FCM-EWT hybrid model with machine learning classifiers.

	SVM			Adaboost			NBC			KNN		
	Sen	Spe	Acc	Sen	Spe	Acc	Sen	Spe	Acc	Sen	Spe	Acc
DE	88.12	89.12	88.62	86.12	85.63	85.875	85.28	84.59	84.935	88.12	87.02	87.57
FCM	89.12	89.23	89.175	88.24	87.23	87.735	87.57	86.89	87.23	86.35	86.78	86.565
EWT	92.21	93.43	92.82	90.24	89.38	89.81	89.49	88.51	89	88.76	89.12	88.94
DE-FCM	93.23	92.23	92.73	91.25	91.01	91.13	90.17	91.28	90.725	90.234	90.23	90.232
DE-EWT	94.24	94.02	94.13	92.16	91.11	91.635	91.01	90.24	90.625	91.23	91.23	91.23
FCM-EWT	96.45	95.91	96.18	94.23	93.13	93.68	92.69	91.23	91.96	93.34	92.98	93.16
DE-FCM-EWT	98.34	98.09	98.215	95.73	94.25	94.99	93.47	93.08	93.275	94.03	93.11	93.57

**Table 4:** Performance analysis of the LMD-fuzzy C-means with combined kernel SVM.

	SVM-linear kernel			SVM-polynomial kernel			Combined kernel LS-SVM		
	Sen	Spe	Acc	Sen	Spe	Acc	Sen	Spe	Acc
LMD	92.23	93.25	92.74	93.23	93.45	93.34	96.54	96.32	96.43
Fuzzy C-means	94.46	95.23	94.845	94.88	94.99	94.935	97.55	97.91	97.73
LMD-fuzzy C-means	96.11	95.12	95.615	96.91	96.03	96.47	98.23	98.78	98.505

Figure 6 illustrates the GDR analysis for the dynamic graph construction and graph entropy-based machine learning technique. It is evident from Figure 6 that a high GDR is found for the proposed dynamic graph construction and graph entropy-based SVM classification technique. Figure 7 illustrates the GDR analysis for dimensionality deduction with the EA-BBN-ELM hybrid model and it is evident that a high GDR is found for the LTSA-based PSO-BBN-ELM hybrid model. Figure 8 illustrates the GDR analysis of the DE-FCM-EWT hybrid model with machine learning classifiers and a high GDR is found for the DE-FCM-EWT model with SVM classifier. Figure 9 illustrates the GDR analysis of the LMD-fuzzy C-means with SVM and combined kernel SVM and a high GDR is observed for the LMD-fuzzy C-means model classified with the combined kernel SVM.

### 3.1. Comparison with previous works

The results of our research have been compared with the results of previous works done on the same dataset. Only one published paper was found in the literature in the year 2022 where the same dataset was used and a result of about 99.17% was obtained by means of applying a MCBP method classified with a SVM classifier (Tuncer et al., 2022). In Tuncer et al. (2022), the authors used just one methodology to obtain that result. However, in our work, four different methods have been proposed encompassing many ideas and an exhaustive analysis has been conducted. The best results were obtained for the DE-FCM-EWT hybrid model with SVM classifier reporting a classification accuracy of 98.21%. The second-best classification accuracy (of 98.5%) was obtained using the LMD-fuzzy C-means clustering technique classified with a combined kernel LS-SVM model. The third best classification accuracy (of 97.57%) was obtained using the LTSA-based EA-BBN-ELM model and the fourth best classification accuracy (of 96.44%) was obtained for the dynamic graph-based construction with graph entropy and SVM classifier. As far as the first proposed strategy is concerned, the lowest classification accuracy (of 85.52%) was obtained when the dynamic graph construction concept was implemented directly with NBC. As far as the second proposed strategy is concerned, the lowest classification accuracy (of 91.72%) was obtained when the LLC with GSO-BBN-ELM model was used. As far as the third proposed strategy is concerned, DE with NBC produced a lower accuracy of 84.93% and, as far as the final proposed strategy is concerned, the LMD with SVM linear kernel produced a lower accuracy of 92.74%. Thus, for different combinations, an exhaustive analysis has been done to analyze the best and worst performing methods and the results are projected clearly.

## 4. Conclusion and future work

A good research area in the field of biomedical engineering is in the field of neuro-prosthetics as it has gained a lot of popularity in the past few decades. Significant advancements in prosthetics control allow amputees to complete more tasks independently, although the classification accuracy remains a huge challenge. sEMG signals are highly useful for the control and application of prosthetic control, where these signals are implemented for robotic control of fingers, arms, and hands. With the advent of automated machine learning techniques, the automated classification of sEMG signals has been explored to a great extent and in our paper four methods have been proposed. The best results are obtained using the DE-FCM-EWT hybrid model with SVM classifier, reporting a classification accuracy of 98.21%, and the second-best classification accuracy (of 98.5%) is obtained using the LMD-fuzzy C-means clustering technique classified with a combined kernel LS-SVM model. In the future, the plan is to implement it on bigger sEMG datasets. Also, the plan is to further implement a variety of other machine learning and transfer learning techniques. Future work is also planned to incorporate advanced deep learning models if bigger datasets are available. In future, the work can also be implemented for telemedicine applications so that remote healthcare monitoring systems can be improved.

## Data availability statement

Publicly available datasets were analyzed in this study. This data can be found here: “R.N. Khushaba, S. Kodagoda, Electromyogram (EMG) feature reduction using mutual components analysis for multifunction prosthetic fingers control, 2012, 12th International Conference on Control Automation Robotics & Vision (ICARCV), IEEE, 2012, pp. 1534-1539”.

## Ethics statement

Ethical review and approval was not required for the study on human participants in accordance with the local legislation and institutional requirements. Written informed consent from the patients/participants or patients/participants’ legal guardian/next of kin was not required to participate in this study in accordance with the national legislation and the institutional requirements.

## Author contributions

SP - concept, methodology, visualization, implementation, paper draft. D-OW - visualization, critical revision, supervision, funding, project management. All authors contributed to the article and approved the submitted version.

## Funding

This work was supported by Institute of Information & Communications Technology Planning & Evaluation (IITP) grant funded by the Korea government (MSIT) (No. 2017-0-00451, Development of BCI based Brain and Cognitive Computing Technology for Recognizing User’s Intentions using Deep Learning) and partly supported by the National Research Foundation of Korea (NRF) grant funded by the Korea government(MSIT) (Nos. 2022R1A5A8019303, 2022R1F1A1074640).

## Conflict of interest

The author declare that the research was conducted in the absence of any commercial or financial relationships that could be construed as a potential conflict of interest.

## Publisher’s note

All claims expressed in this article are solely those of the authors and do not necessarily represent those of their affiliated organizations, or those of the publisher, the editors and the reviewers. Any product that may be evaluated in this article, or claim that may be made by its manufacturer, is not guaranteed or endorsed by the publisher.
